# Rapid Screening of Potential Phosphodiesterase Inhibitors from the Roots of* Ilex pubescens* Hook. et Arn. Using a Combination of Ultrafiltration and LC–MS

**DOI:** 10.1155/2017/2749643

**Published:** 2017-03-23

**Authors:** Zichen Liu, Zongtao Lin, Shizhong Chen, Lingjun Wang, Shaoxiang Xian

**Affiliations:** ^1^Guangzhou Key Laboratory of Chinese Medicine Prevention and Treatment of Chronic Heart Failure, The First Affiliated Hospital, Guangzhou University of Chinese Medicine, Guangzhou, Guangdong 510407, China; ^2^Department of Pharmaceutical Sciences, University of Tennessee Health Science Center, Memphis, TN 28163, USA; ^3^School of Pharmaceutical Sciences, Peking University, Beijing 100191, China

## Abstract

The cyclic nucleotide phosphodiesterase (PDE) plays an important role in regulating the levels of second messenger molecules cAMP and cGMP. Various PDE inhibitors have been successfully developed into drugs for targeted diseases. In addition, PDE inhibitors can also be found in different foods and natural medicines. In this study, ultrafiltration liquid chromatography–diode-array detector–electrospray ionization–ion-trap–time-of-flight–mass spectrometry (ultrafiltration LC–DAD–ESI–IT–TOF–MS) was applied to screen PDE inhibitors from the roots of* Ilex pubescens* Hook. et Arn. As a result, 11 major compounds were identified in* I. pubescens *roots, with nine compounds as potential PDE inhibitors, among which five were further confirmed to be active against PDEI and PDE5A dose-dependently in vitro, with ilexsaponin A_1_ and ilexsaponin B_2_ being the strongest. HPLC quantification of these bioactive compounds suggested that they are major components in the plant. The results demonstrate that ultrafiltration LC–DAD–ESI–IT–TOF–MS is an efficient method for rapid screening of PDE inhibitors from natural medicines.

## 1. Introduction

Phosphodiesterase (PDE) hydrolyzes phosphodiesters to phosphomonoesters and is expressed in various tissues and organs intracellularly and extracellularly [1]. The cyclic nucleotide phosphodiesters, both cAMP and cGMP, play important roles in cardiac physiology and pathology and can be hydrolyzed specifically by PDEs [2–4]. In addition, seven of the 11 PDE family members are known to be expressed in the heart tissue, suggesting the importance of PDEs to the cardiac system. PDEs regulate many crucial processes maintaining cardiac function and other pathological signaling pathways [5]. Among PDEs, PDE5 is cGMP-activated and cGMP-specific and has been a crucial target for developing treatments of cardiovascular diseases [2]. Some successful PDE5 inhibitors such as Sildenafil, Tadalafil, and Vardenafil have already been approved for erectile dysfunction and pulmonary hypertension [3, 6–8]. Encouraged by the success and broad applications of PDE inhibitors, investigations on the novel types of PDE inhibitors have drawn increasing attentions of researchers.

*Ilex pubescens* Hook. et Arn. (*Maodongqing* in Chinese) is well known for its roots, which are used as a traditional Chinese medicine for the treatment of cardiovascular diseases such as coronary arterial thrombosis [9, 10], heart failure [11], stroke [12], and thromboangiitis obliterans [13] for a long time. In addition, the leaves of* I. pubescens *are a herbal tea [14] and* Radix Ilicis Pubescentis *can be used as an ingredient for functional food or soup.* I. pubescens* contains various chemical components including flavonoids, triterpene saponines, lignans, and phenolic acids [15]. Our previous study has shown that* Radix Ilicis Pubescentis *extract dose-dependently improved the cardiac function and ventricular remodeling on rats with chronic heart failure [16]. And our preliminary study additionally revealed that the extract from* Radix Ilicis Pubescentis *showed potent PDE inhibitory activities. However, the active compounds responsible for these in vitro and in vivo activities in the extract are still unclear.

Ultrafiltration provides centrifugal force and a semipermeable membrane to separate solids and high-molecular-weight solutes from liquid and low-molecular-weight solutes. Combined with LC–MS which has become a powerful technique for simultaneous separation and identification of components, ultrafiltration LC–MS has become the state-of-the-art technique in screening and identifying active constituents in complex extracts, owing to the capability of separation of ligand-protein complexes from unbound compounds by ultrafiltration and the capability of identification of the ligands by LC–MS [17–19].

In this study, the chemical basis for the PDE inhibitory activity of* I. pubescens *roots was revealed for the first time. Nine of eleven components in* I. pubescens *roots were identified as PDE binders by the established ultrafiltration LC–MS method, five of which were further confirmed to be PDE inhibitors by in vitro inhibitory assay and quantified by HPLC. This work suggested that* I. pubescens *roots contained health beneficial compounds and can be potentially used as a functional source of medicine, soup, and/or food for the improvement of cardiac diseases.

## 2. Experimental

### 2.1. Materials and Reagents

Roots of* I. pubescens *were purchased from Guangzhou Zisun Pharmaceutical Co. Ltd. and were authenticated by Professor Shizhong Chen in the Department of Natural Medicines, Peking University. Reference powder of roots of* I. pubescens* was purchased from the National Institute for Food and Drug Control (Beijing, China). Ilexgenin A, ilexsaponin A_1_, ilexsaponin B_1_, ilexsaponin B_2_, chlorogenic acid, isochlorogenic acid B, and isochlorogenic acid C were obtained from Chengdu Jioute Biological Technology Co. Ltd. (Chengdu, China). The purity of each compound was determined by HPLC as above 98%. Phosphodiesterase I (PDEI) from* Crotalus adamanteus* venom (130 U/mg) was purchased from Shanghai Yuanye Bio Technology Co., Ltd. (Shanghai, China). Recombinant human PDE5A was obtained from Enzo Life Sciences, Inc. (New York, USA).

HPLC grade acetonitrile, LC–MS grade acetonitrile, and formic acid (FA) were bought from Fisher Scientific (Geel, Belgium). Analytical grade methanol (Beijing Chemical Works, Beijing, China) was used for sample preparation. Deionized water (18.2 M*Ω*) was prepared by a Milli-Q system (Millipore, MA, USA). Amicon® Ultra-4 10K centrifugal filter devices with maximum initial sample volume of 4.0 mL (Millipore, MA, USA) were used for sample ultrafiltration.

### 2.2. Sample Preparation

Roots of* I. pubescens* were pulverized into homogenized powder (number 80 mesh sieve), 5.0 g of which was accurately weighed and extracted with 50 mL of methanol in an ultrasonic water bath for 60 min. After centrifuging for 10 min at 8000 rpm, the supernatant was collected and dried by rotavapor under reduced pressure. The residue was dissolved in methanol with a concentration of 0.1 g·mL^−1^ (in terms of raw material). The solution was filtered through a 0.22 *μ*m membrane readily for injection (5 *μ*L) for LC–MS analysis.

### 2.3. Separating PDE Inhibitors from* I. pubescens* Roots by Ultrafiltration

The above-mentioned sample (120 *μ*L) was diluted to 1.0 mL with 100 mM HEPES (150 mM NaCl, pH 7.4) and was then incubated with 1.0 mL of 10 U·mL^−1^ and 20 U·mL^−1^ PDEI in 100 mM HEPES (150 mM NaCl, pH 7.4) at 37°C for 30 min, respectively. The mixture was filtered by a centrifugal ultrafiltration device (Amicon Ultra-4 10K, cut-off molecular weight: 10 kDa) at 7500 ×g for 20 min at 25°C. The ligand-enzyme complexes on the filter were washed with 100 mM HEPES (150 mM NaCl, pH 7.4) (3 × 1 mL) by ultrafiltration. The filter was then washed with 50% methanol (3 × 0.5 mL), which was collected and dried. The residue was dissolved in 200 *μ*L methanol, filtered through a 0.22 *μ*m membrane before injection (20 *μ*L) for LC–MS analysis. Solution obtained without enzyme was used as a negative control. The binding assays were carried out in triplicate (*n* = 3).

HPLC peak area enhanced as a result of incubation, which indicates binding of a ligand to PDEI. The enhancement factor (%) = (*A*_1_/*A*_2_) × 100% was used for calculating the binding of a ligand to PDEI, where *A*_1_ and *A*_2_ are the amounts of compound bound to PDEI and the total amount of compound in incubation, respectively [17, 20].

### 2.4. Structural Characterization by LC–DAD–ESI–IT–TOF–MS

#### 2.4.1. LC Conditions

Chromatographic analysis was performed on a Shimadzu HPLC system (Kyoto, Japan) including two LC-10AD pumps, a CTO-10ASvp column oven, a DGU-14A degasser, an SIL-HTC autoinjector, and an SPD-M10Avp DAD detector. Separation was carried out on an Inertsil ODS-4 C_18_ column (5 *μ*m, 250 mm × 4.6 mm, Shimadzu, Kyoto, Japan) maintained at 30°C. Linear-gradient elution using mobile phases A (water containing 0.1% FA) and B (acetonitrile containing 0.1% FA) was as follows: 0–2 min, 5% B; 2–40 min, 5% → 30% B; 40–60 min, 30% → 40% B; 60–75 min, 40% → 55% B; 75–85 min, 55% → 85% B; 85–90 min, 85% → 90% B. The flow rate was 1.0 mL·min^−1^. The online UV spectra were recorded in the range of 200–400 nm, and the DAD was set at 327 nm for 0–50 min and at 210 nm for 50–90 min.

All calibration solutions were prepared by serial dilution of the individual stock solutions of the standards with methanol and filtered through a 0.22 *μ*m membrane filter before HPLC analysis. 5 *μ*L of each solution was injected into the HPLC system in triplicate, and a calibration curve was generated between the HPLC peak areas of compound and the concentrations for content determination. Peaks are integrated automatically by LC–MS solution software (version 3, Shimadzu, Kyoto, Japan), and the correlation coefficients were obtained using the linear regression model in Excel® (Microsoft) with all above 0.9997.

#### 2.4.2. MS Conditions

MS analysis was performed on an IT–TOF mass spectrometer (Shimadzu LC–MS–IT–TOF, Kyoto, Japan) with an ESI interface. A full-scan MS reading in both negative and positive ion modes over the* m/z* range 100–1100 was performed. The CDL temperature and block heater temperature were both 200°C. The capillary voltage, CDL voltage, and detector voltage were set at 4.5 kV, 10 V, and 1.7 kV, respectively. The flow rate of nebulizer gas (N_2_) was adjusted to 1.5 L·min^−1^. During HPLC–MS analysis, the collision energy was set to 70% and the isolation width of precursor ions was 3.0 U. LC–MS solution software (version 3, Shimadzu, Kyoto, Japan) was used for data acquirement and processing.

### 2.5. PDE Inhibition Assay

The PDE inhibitory assay was carried out spectrophotometrically using Cyclic Nucleotide Phosphodiesterase Assay Kit (A BIOMOL® GREEN Quantizyme® Assay System, Enzo Life Sciences, Inc., New York, USA). The PDE inhibition activity was calculated as follows: inhibition (%) = (*A*_1_ − *A*_2_)/*A*_2_ × 100, where *A*_1_ is the absorbance of the control and *A*_2_ is the absorbance of the sample. The inhibitory activity was shown as the sample concentration needed to inhibit 50% of the enzymatic activity (IC_50_).

## 3. Results and Discussion

### 3.1. LC Analysis of PDE Inhibitors after Ultrafiltration

**Figure 1:**
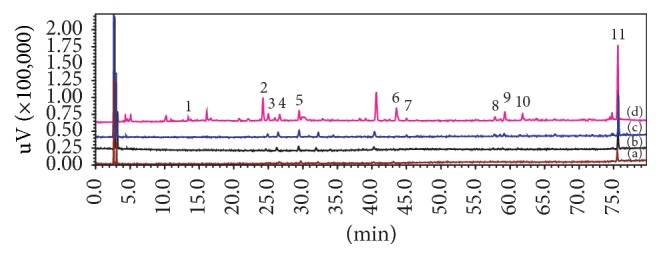
Ultrafiltration HPLC chromatograms of potential PDE inhibitors in* I. pubescens* roots. PDE I concentration: (a) 0 U·mL^−1^; (b) 10 U·mL^−1^; (c) 20 U·mL^−1^; (d) crude extract of* I. pubescens* roots.

The ultrafiltration LC–MS method can be used for the screening of bioactive compounds from complex mixtures, especially for natural products with a minimal amount of sample [21]. When the extract of* I. pubescens *roots was incubated with PDEI, potential inhibitors bound to the enzyme and unbound small molecules can be separated from the ligand-PDE complexes or PDEI by the ultrafiltration membrane. In this way, the ultrafiltration device holds active compounds bound with the receptor, while filtrating out free molecules. The binding will be affected by both the potential inhibitor and receptor concentrations. The ratio of PDEI enzyme to the root extract should be low to keep all enzyme molecules saturated by enzyme binders, while the amount of enzyme should be sufficient to absorb enough compounds for HPLC analysis. In addition, the use of highly concentrated enzyme (>50 U·mL^−1^) would be a waste of PDEI material. In this study, the effect was evaluated by incubating a fixed concentration of the extract (0.1 g·mL^−1^, in terms of raw material, 0.120 mL in volume) with two levels of PDEI (10 U·mL^−1^ and 20 U·mL^−1^, 1 mL in volume) optimized from the previous study [21]. Only compounds with certain binding ability to PDEI will be detected by LC analysis after ultrafiltration. Applying this method, nine of 11 major compounds of* I. pubescens *roots were identified as potential PDE inhibitors.  Figure 1 shows the chromatograms of ultrafiltration LC analysis of* I. pubescens *root extract.

### 3.2. Identification of 11 Major Compounds in* I. pubescens* Roots

**Figure 2:**
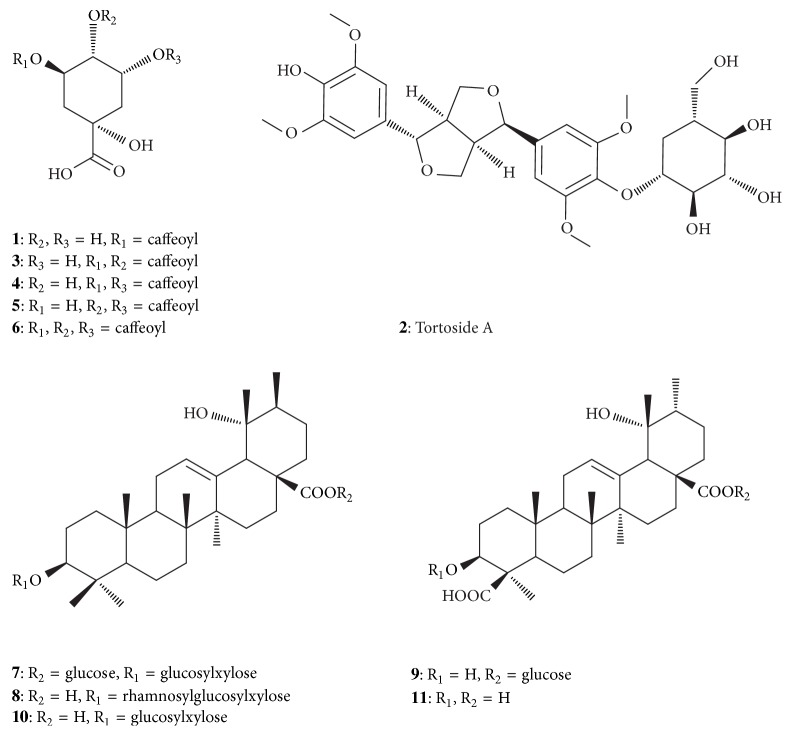
Structures of the 11 compounds identified in* Radix Ilicis Pubescentis*.

LC–PDA–ESI–IT–TOF–MS analysis was used to identify the 11 major compounds in* I. pubescens* roots. The mass spectral data in negative ion mode was used for characterization. The MS fragmentations of compounds together with their retention times (*t*_*R*_) are summarized in Table 1. Their structures were shown in Figure 2. The ultrafiltrate was analyzed under the same condition. The retention times and MS data (see Table S1 in the Supplementary Material available online at https://doi.org/10.1155/2017/2749643) of the 11 compounds obtained from the ultrafiltrate were identical to those from* I. pubescens *roots extract. The EIC chromatograms of each compound in negative ion mode of the extract, ultrafiltrate, and blank sample were also shown in supplementary Figures S1–9. Compounds** 1**,** 3**,** 5**,** 8**,** 9**,** 10**, and** 11** were further identified unambiguously by comparing their chromatographic and MS behaviors to corresponding standard compounds as shown in Table S1.

Peak** 1** gave [M-H]^−^ signal at* m/z* 353.0882 (C_16_H_17_O_9_, error = 2.5 ppm) and a fragment ion at* m/z* 191 indicating the loss of caffeic acid [22–24]. It was therefore identified as chlorogenic acid [25]. Peak** 2** yielded an [M-H]^−^ ion at 579.2096 (C_28_H_35_O_13_, error = 3.1 ppm) and a product ion at 417 ([M-H-Glc]^−^) indicated the loss of a glucosyl (Glc) moiety, which further yielded an ion* m/z* 181 (syringyl) owing to the *α*,*β*-cleavage of the phenolic ether [26]. According to previous data [27], compound** 2** was tentatively characterized as tortoside A. Peaks** 3**,** 4**, and** 5** showed similar retention times (Table 1) and gave their [M-H]^−^ ions at* m/z* 515.1230, 515.1190, and 515.1215 (C_25_ H_23_O_12_, 515.1190), respectively. The ions at* m/z* 179 and 191 in negative mode were deprotonated ions of caffeic acid ([CA-H]^−^) and quinic acid ([QA-H]^−^). Compared with standard compounds and previous reports [25, 28–32], peaks** 3**,** 4**, and** 5** were thus identified as isomers of dicaffeoylquinic acid, isochlorogenic acid B, isochlorogenic acid A, and isochlorogenic acid C, respectively.

The [M-H]^−^ ions of peaks** 8** and** 10** were at* m/z* 911.5049 (C_47_H_75_O_17_, error = 4.9 ppm) and* m/z* 765.4400 (C_41_H_65_O_13_, error = −3.3 ppm), respectively. They both showed* m/z* 603 in MS^2^ which displayed the same fragmentation pattern in MS^3^, indicating their structural similarity. Compared with standards, compounds** 8** and** 10** were unambiguously identified as ilexsaponin B_1_ and ilexsaponin B_2_ [33]. Peaks** 9** and** 11 **gave [M-H]^−^ ions at* m/z* 663.3766 (C_36_H_55_O_11_, error = 3.3 ppm) and* m/z* 501.3239 (C_30_H_45_O_6_, error = 4.6 ppm), respectively. They shared similar fragmentation behaviors of their common ion at* m/z* 501. They were identified as ilexsaponin A_1_ and ilexgenin A, respectively, after comparison with standard compounds and literature report [34].

Peak** 7** exhibited its [M-H]^−^ ion at* m/z* 927.4996 (C_47_H_75_O_18_, error = 4.6 ppm) and [M+HCOO]^−^ ion at* m/z* 973.5043 (C_48_H_77_O_20_, error = 3.6 ppm). The ion at* m/z *765 and 603 indicated the losses of two Glc moieties from the structure. According to the previous report [33], compound** 7** was tentatively characterized as ilexsaponin B_3_. Peak** 6** showed an [M-H]^−^ signal at* m/z* 677.1533 (C_34_H_29_O_15_, error = 3.8 ppm) and yielded a product ion at* m/z* 515 indicating the loss of a caffeoyl group. This ion further produced ions at* m/z* 353 ([M-H-2caffeoyl]^−^) and 191 ([M-H-3caffeoyl]^−^) indicating caffeoyl substitutions on the quinic acid skeleton. In addition, its base peak at* m/z* 173 ([QA-H-H_2_O]^−^) and ions at* m/z* 179, 191, and 135 in MS^4^, along with the presence of the fragment ion* m/z* 335 in MS^3^, were identical to those of 3,4-dicaffeoylquinic acid [35]. Compared to the previous reports, compound** 6** was thus tentatively characterized as 3,4,5-tricaffeoylquinic acid [36, 37].

### 3.3. Validation of the PDE Inhibition Activity of Identified Compounds

#### 3.3.1. PDE Inhibitory Activity of Active Compounds In Vitro

To validate the activities of the identified compounds, in vitro inhibitory assays against PDEI and PDE5A were conducted using six selected compounds [isochlorogenic acid B (**3**), isochlorogenic acid C (**5**), ilexsaponin B_2_ (**8**), ilexsaponin A_1_ (**9**), ilexsaponin B_1_ (**10**), and ilexgenin A (**11**)]. Their IC_50_ against PDEI were 779.5, 0, 853.7, 477.5, 332.0, and 837.7 *μ*M, while those of PDE5A were 193.5, 0, 48.8, 22.4, 1801.7, and 176.6 *μ*M, respectively, suggesting their selectivities against PDEI and PDE5A (Table 2). Dose-dependent effects were observed for all compounds except inactive isochlorogenic acid C.

#### 3.3.2. Relative Binding Efficiency with PDEI Expressed by the Enhancement Factor

The enhancement factor (Table 2) was introduced to further understand the relative binding strength of each compound [17, 20]. This factor is proportional to the LC peak area of the ligand bound to the target enzyme; thus the higher factor means more bound compound. Although ilexgenin A is a predominant peak (peak** 11**) and with a concentration of 8.96 mg/g in the* Radix Ilicis Pubescentis* (Figure 1(d) and Table 2), its enhancement factor is less than that of ilexsaponin B_2_ (**8**) which is the strongest PDEI ligand with a concentration of 1.24 mg/g in the sample (Figure 1(a–c)). Differently, ilexsaponin A_1_ (**9**) with low binding efficiency turned out to be the strongest PDE inhibitor (332.0 *μ*M and 22.4 *μ*M against PDEI and PDE5A, resp.) among the components tested in vitro. Interestingly, isochlorogenic acid C (**5**) showed quite strong bindings to PDEI, but it was not active against PDEI and PDE5A (Table 2) indicating unspecific binding between isochlorogenic acid C and PDEI.

The validation results suggested that the ultrafiltration LC–MS is an efficient tool to screen PDE-binding active compounds, although their binding degrees did not perfectly match with their IC_50_ values in vitro. Ilexsaponin A_1_ and ilexsaponin B_2_ showed stronger inhibitory activity than other constituents, suggesting that they should be considered for quality control purposes of* I. pubescens *roots for pharmaceutical use or as a functional food source.

## 4. Conclusion

In this study, an ultrafiltration LC–MS method was established for rapid screening potential PDE inhibitors for the first time. Nine of eleven compounds from* Radix Ilicis Pubescentis *were identified as PDE-binding active compounds by this method. Further in vitro assays confirmed the PDE inhibitory activity of components in* Radix Ilicis Pubescentis*. It is worth noting that the ultrafiltration screening method is based on the binding affinity, and the PDE binders are not necessarily the PDE inhibitors. The strong inhibitory effects of ilexsaponin A_1_ and ilexsaponin B_2_ against PDE5A provide further understanding of the beneficial effects of* I. pubescens *roots on cardiovascular system and also suggest that active products from natural plants/medicines can be health beneficial as edible and functional food sources. The success of this study suggested that the established approach could be a valuable tool for rapid screening of PDE inhibitors from* I. pubescens *and other complex samples.

## Supplementary Material

The retention times and MS data of the extract, ultrafiltrate and standard compound were summarized (Table S1). The EIC chromatograms of each compound in negative ion mode of the extract, ultrafiltrate, and blank sample were shown (Figures S1–9).

## Figures and Tables

**Table 1:** Characterization of constituents by LC–ESI–IT–TOF–MS from the roots of *I. pubescens*.

Number	*t*_*R*_ (min)	Formula (theoretical* m/z*)	Experimental *m/z* (error, ppm)	Fragmentation	Identification
**1**	12.312	C_16_ H_18_ O_9_ (353.0873)	353.0882 [M-H]^−^ (2.5)	353 (100%) → 191 ([M-H-Caffeoyl]^−^)	Chlorogenic acid^*∗*^

**2**	25.468	C_28_H_36_O_13_ (579.2078)	579.2095 [M-H]^−^ (2.9)	579 (100%) → 417 ([M-H-Glc]^−^, 100%) → 402 ([M-H-Glc-CH_3_]^−^), 181 ([Syringyl]^−^)	Tortoside A
			625.2131 [M+HCOO]^−^		

**3**	25.630	C_25_H_24_O_12_ (515.1190)	515.1230 [M-H]^−^ (7.8)	515 (100%) → 353 ([M-H-Caffeoyl]^−^, 100%) → 173 ([M-H-2Caffeoyl-H_2_O]^−^), 179 ([Caffeic acid-H]^−^), 135 ([Caffeic acid-H-CO_2_]^−^)	Isochlorogenic acid B^*∗*^

**4**	27.838	C_25_H_24_O_12_ (515.1190)	515.1190 [M-H]^−^ (0.0)	515 (100%) → 353 ([M-H-Caffeoyl]^−^, 100%), 191 ([Quinic acid-H]^−^) → 191 ([M-H-2Caffeoyl]^−^)	Isochlorogenic acid A

**5**	29.895	C_25_H_24_O_12_ (515.1190)	515.1215 [M-H]^−^ (4.9)	515 (100%) → 353 ([M-H-Caffeoyl]^−^, 100%), 173 ([M-H-2Caffeoyl -H_2_O]^−^) → 173 ([M-H-2Caffeoyl -H_2_O]^−^)	Isochlorogenic acid C^*∗*^

**6**	41.972	C_34_H_30_O_15_ (677.1507)	677.1533 [M-H]^−^ (3.8)	677 (100%) → 515 ([M-H-Caffeoyl]^−^, 100%), 353 ([M-H-2Caffeoyl]^−^) → 353 ([M-H-2Caffeoyl]^−^, 100%), 335 ([M-H-2Caffeoyl- H_2_O]^−^) → 173 ([Quinic acid-H- H_2_O]^−^), 179 ([Caffeic acid-H]^−^), 191 ([Quinic acid-H]^−^), 135 ([Caffeic acid-H-CO_2_]^−^)	3,4,5-Tricaffeoylquinic acid

**7**	45.220	C_47_H_76_O_18_ (927.4953)	927.4996 [M-H]^−^ (4.6)	927 (100%) → 765 ([M-H-Glc]^−^, 100%) → 603 ([M-H-2Glc]^−^)	Ilexsaponin B_3_
			973.5043 [M+HCOO]^−^		

**8**	56.657	C_47_H_76_O_17_ (911.5004)	911.5049 [M-H]^−^ (4.9)	911 (100%) → 765 ([M-H-Rha]^−^, 100%), 603 ([M-H-GlcRha]^−^) → 603 ([M-H-GlcRha]^−^, 100%) → 543 ([M-H-GlcRha-H_2_O-CO_2_]^−^), 585 ([M-H-GlcRha-H_2_O]^−^)	Ilexsaponin B_2_^*∗*^
			957.5107 [M+HCOO]^−^		

**9**	57.873	C_36_H_56_O_11_ (663.3744)	663.3766 [M-H]^−^ (3.3)	663 (100%) → 501 ([M-H-Glc]^−^, 100%) → 483 ([M-H-Glc-H_2_O]^−^, 100%) → 439 ([M-H-Glc-H_2_O-CO_2_]^−^)	Ilexsaponin A_1_^*∗*^
			709.3860 [M+HCOO]^−^		

**10**	60.828	C_41_H_66_O_13_ (765.4425)	765.4400 [M-H]^−^ (−3.3)	765 (100%) → 603 ([M-H-Glc]^−^, 100%) → 585 ([M-H-Glc-H_2_O]^−^), 543 ([M-H-Glc-H_2_O-CO_2_]^−^)	Ilexsaponin B_1_^*∗*^
			811.4497 [M+HCOO]^−^		

**11**	75.675	C_30_H_46_O_6_ (501.3216)	501.3239 [M-H]^−^ (4.6)	501 (100%) → 483 ([M-H-H_2_O]^−^), 439 ([M-H-H_2_O-CO_2_]^−^)	Ilexgenin A^*∗*^

^*∗*^Compound compared with standard. Glc, glucosyl; Rha, rhamnosyl.

**Table 2:** PDE inhibitory activity (IC_50_) and binding effect with PDEI expressed in LC signal enhancement factor of major components of *I. pubescens *roots.

Compound	Inhibitory activity (IC_50_, *μ*M)		Enhancement factor with PDEI (%)^a^		Contents (mg/g)^b^
	PDEI	PDE5A	10 U/mL	20 U/mL	
Isochlorogenic acid B (**3**)	779.5	193.5	19.15 ± 0.77	25.90 ± 1.30	0.23
Isochlorogenic acid C (**5**)	—	—	26.73 ± 0.41	40.03 ± 0.14	0.27
Ilexsaponin B_2_ (**8**)	477.5	48.8	28.73 ± 0.55	41.85 ± 0.86	1.24
Ilexsaponin A_1_(**9**)	332.0	22.4	11.68 ± 0.31	18.59 ± 0.65	2.53
Ilexsaponin B_1_ (**10**)	853.7	1801.7	9.20 ± 0.62	15.54 ± 0.21	2.18
Ilexgenin A (**11**)	837.7	176.6	23.08 ± 0.44	30.82 ± 0.31	8.96

^a^Enhancement factor (%) = (amount of compound specifically bound)/(total amount of compound in incubation) × 100. ^b^Concentration of individual compounds expressed as mg/g of dry plant powder.
