# Supplementary material for: Rapid Screening of Potential Phosphodiesterase Inhibitors from the Roots of Ilex pubescens Hook. et Arn. Using a Combination of Ultrafiltration and LC–MS
Source: Evid Based Complement Alternat Med. 2017 Mar 23;2017:2749643. doi: 10.1155/2017/2749643 (PMC5382325; doi:10.1155/2017/2749643)
Supplement: Supplementary file 1 — The retention times and MS data of the extract, ultrafiltrate and standard compound were summarized (Table S1). The EIC chromatograms of each compound in negative ion mode of the extract, ultrafiltrate, and blank sample were shown (Figures S1–9). [file 2749643.f1.pdf]

Supplementary data

Table S1. LC–MS data comparison of *I. pubescens* roots extract and ultrafiltrate.

No.	Formula	Compound	Theoretical ([M-H]$^-$)	t_R(min)			Experimental *m/z* (error)		
				extract	ultrafiltrate	Standard	extract	ultrafiltrate	Standard
**1**	$C_{16}H_{18}O_9$	Chlorogenic acid	353.0873	12.312	12.505	12.348	353.0882 (2.5 ppm)	353.0883 (2.8 ppm)	353.0874 (0.3 ppm)
**2**	$C_{28}H_{36}O_{13}$	Tortoside A	579.2078	25.468	25.467		579.2095 (2.9 ppm)	579.2040 (-6.4 ppm)	
**3**	$C_{25}H_{24}O_{12}$	Isochlorogenic acid B	515.1190	25.630	25.622	25.630	515.1230 (7.8 ppm)	515.1199 (1.7 ppm)	515.1212 (4.3 ppm)
**4**	$C_{25}H_{24}O_{12}$	Isochlorogenic acid A	515.1190	27.838	27.653		515.1190 (0.0 ppm)	515.1163 (-5.2 ppm)	
**5**	$C_{25}H_{24}O_{12}$	Isochlorogenic acid C	515.1190	29.895	29.787	29.643	515.1215 (4.9 ppm)	515.1189 (-0.2 ppm)	515.1194 (0.8 ppm)
**6**	$C_{34}H_{30}O_{15}$	3,4,5-Tricaffeoylquinic acid	677.1507	41.972	41.710		677.1533 (3.8 ppm)	677.1494 (-1.9 ppm)	
**7**	$C_{47}H_{76}O_{18}$	Ilexsaponin B_3	927.4953	45.220	45.182		927.4996 (4.6 ppm)	927.4965 (1.3 ppm)	
**8**	$C_{47}H_{76}O_{17}$	Ilexsaponin B_2	911.5004	56.657	56.435	56.463	911.5049 (4.9 ppm)	911.4976 (-3.1 ppm)	911.4960 (-4.8 ppm)
**9**	$C_{36}H_{56}O_{11}$	Ilexsaponin A_1	663.3744	57.873	57.787	57.868	663.3766 (3.3 ppm)	663.3747 (0.5 ppm)	663.3709 (-5.3 ppm)
**10**	$C_{41}H_{66}O_{13}$	Ilexsaponin B_1	765.4425	60.828	60.597	60.572	765.4400 (-3.3 ppm)	765.4444 (2.5 ppm)	765.4437 (1.6 ppm)
**11**	$C_{30}H_{46}O_6$	Ilexgenin A	501.3216	75.675	75.628	75.567	501.3239 (4.6 ppm)	501.3227 (2.2 ppm)	501.3195 (-4.2 ppm)

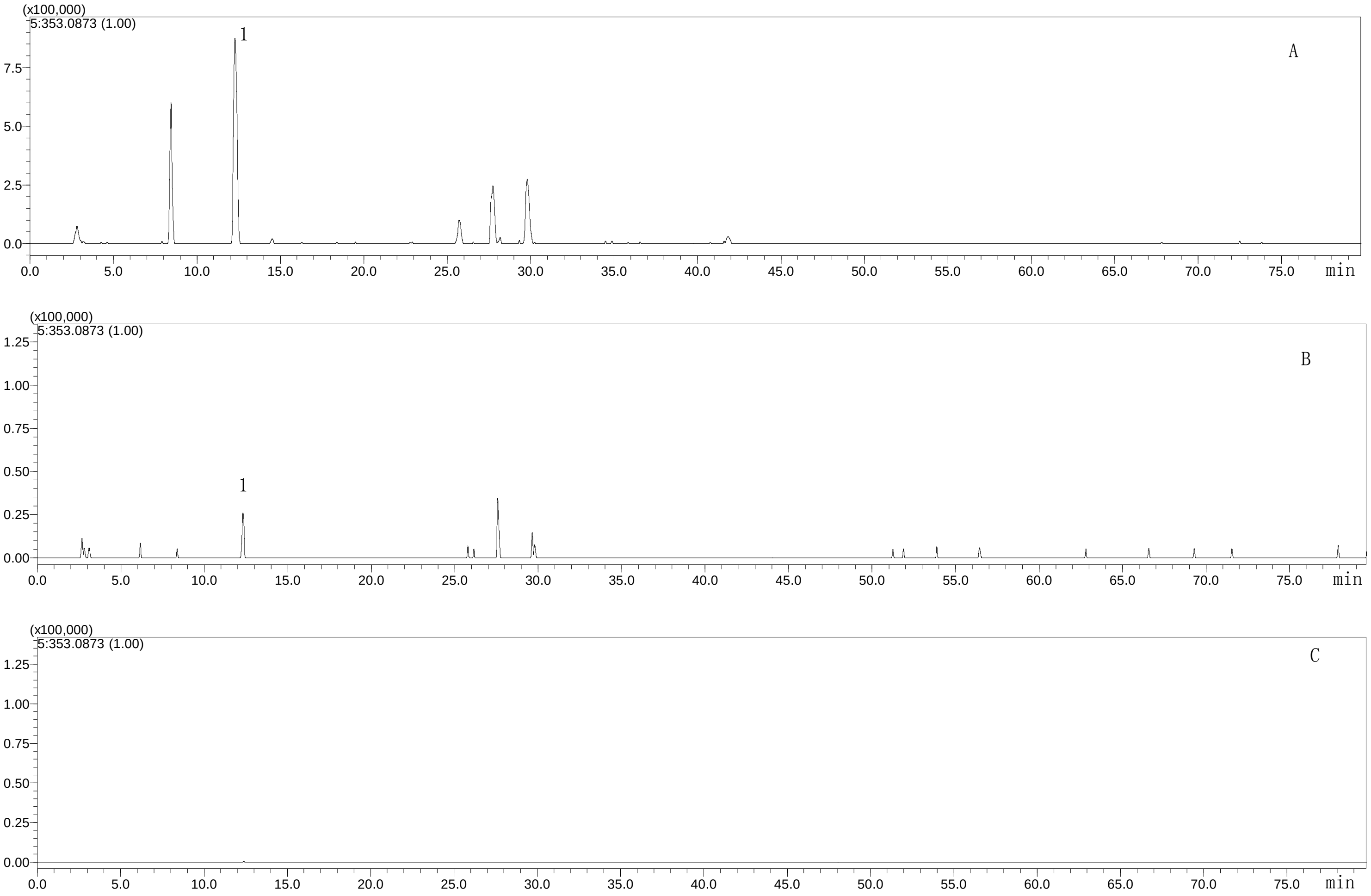

Figure S1. EIC chromatograms of compound **1** in negative mode at *m/z* 353.0873 from *I. pubescens* roots extract (A), ultrafiltrate (B) and blank sample (C).

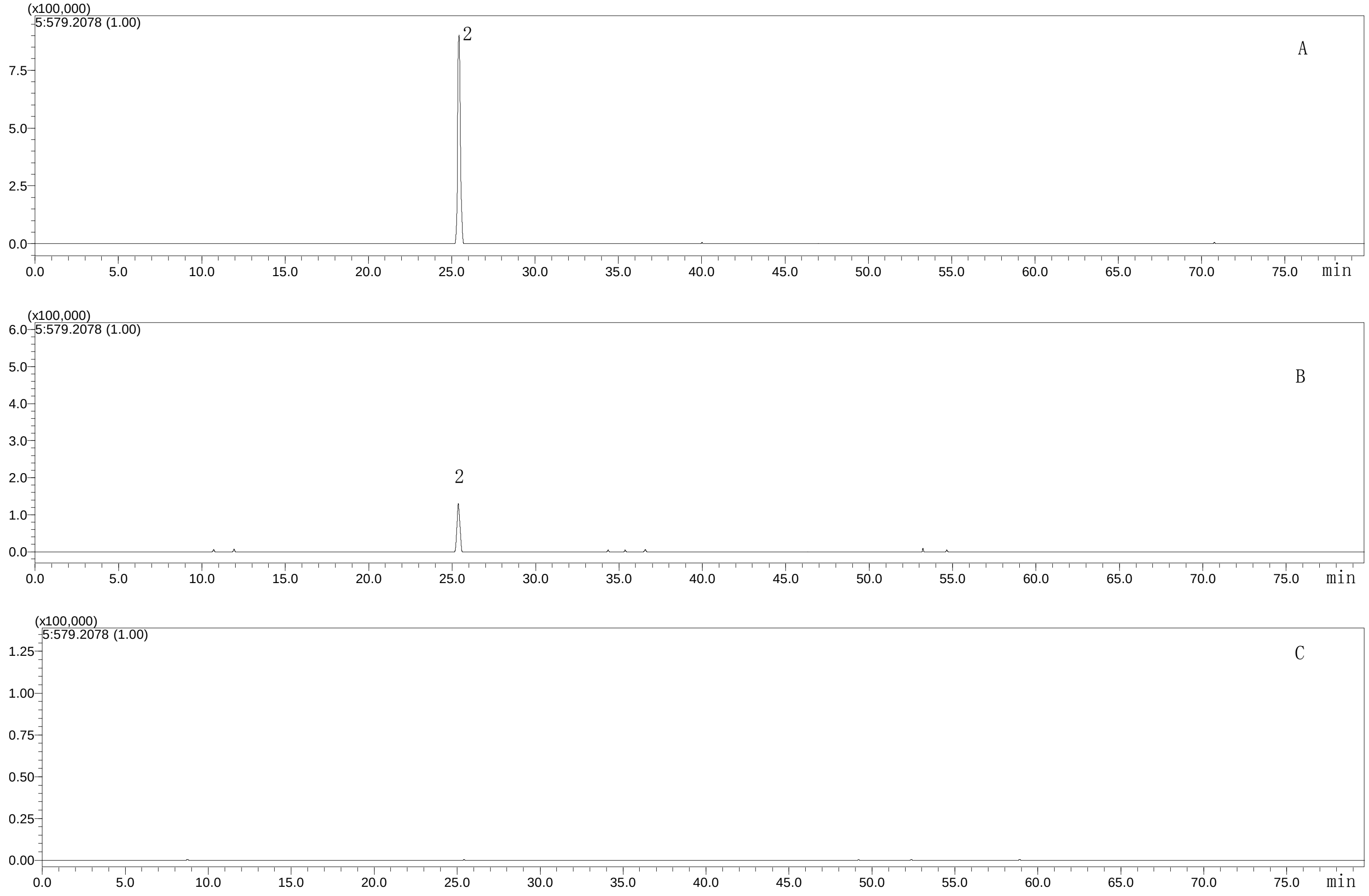

Figure S2. EIC chromatograms of compound **2** in negative mode at *m/z* 579.2078 from *I. pubescens* roots extract (A), ultrafiltrate (B) and blank sample (C).

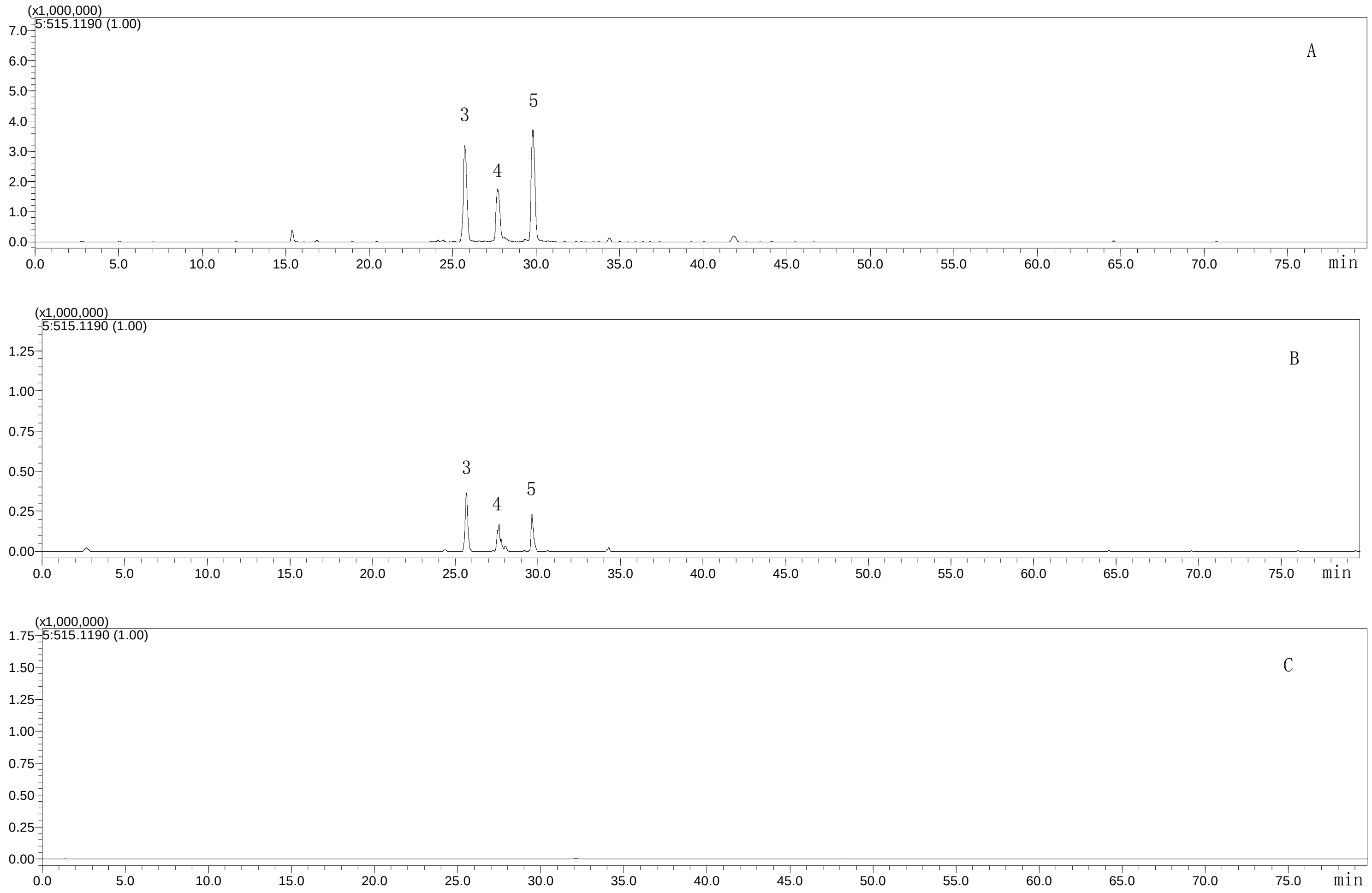

Figure S3. EIC chromatograms of compound **3, 4, 5** in negative mode at *m/z* 515.1190 from *I. pubescens* roots extract (A), ultrafiltrate (B) and blank sample (C).

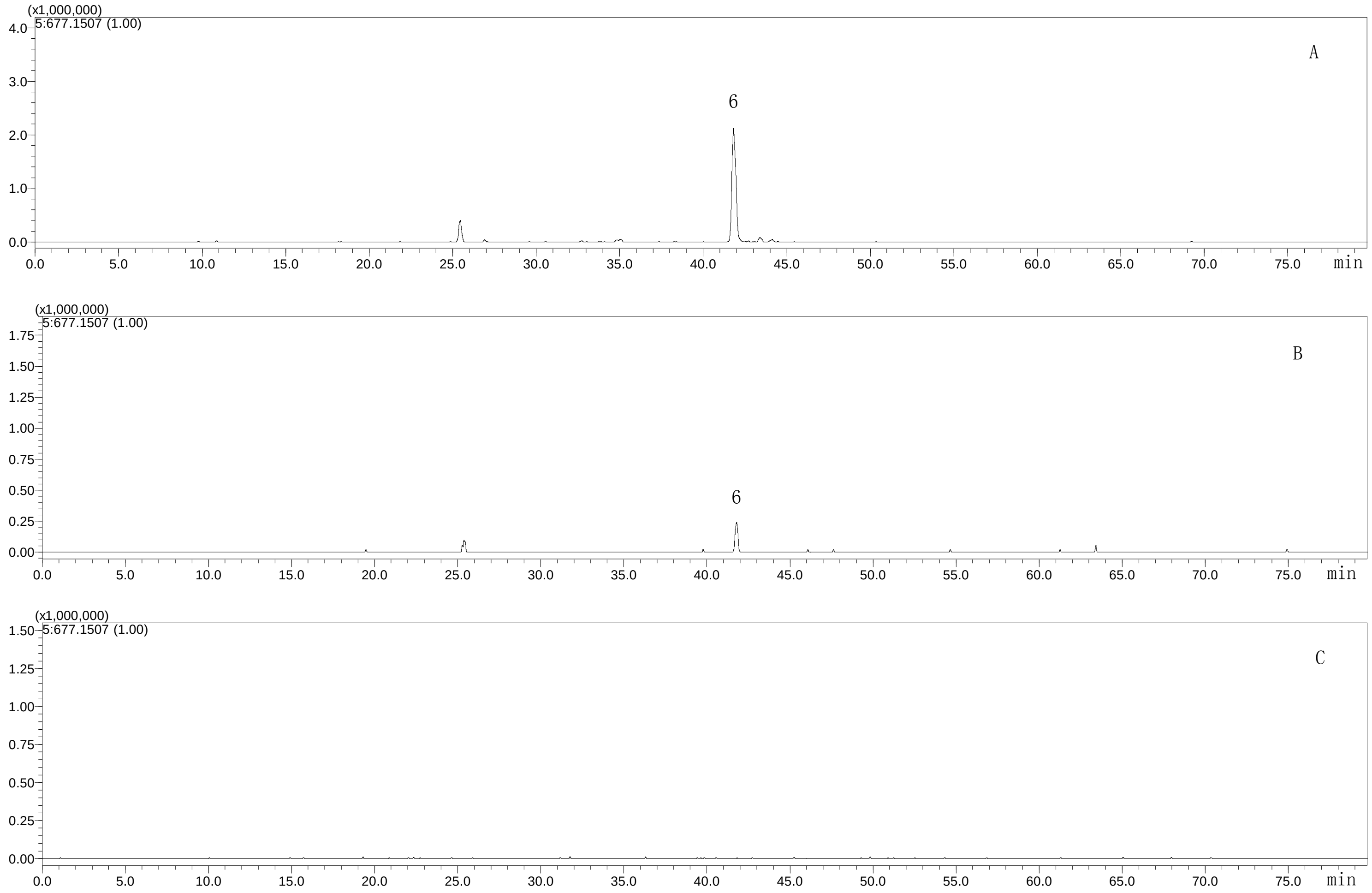

Figure S4. EIC chromatograms of compound **6** in negative mode at *m/z* 677.1507 from *I. pubescens* roots extract (A), ultrafiltrate (B) and blank sample (C).

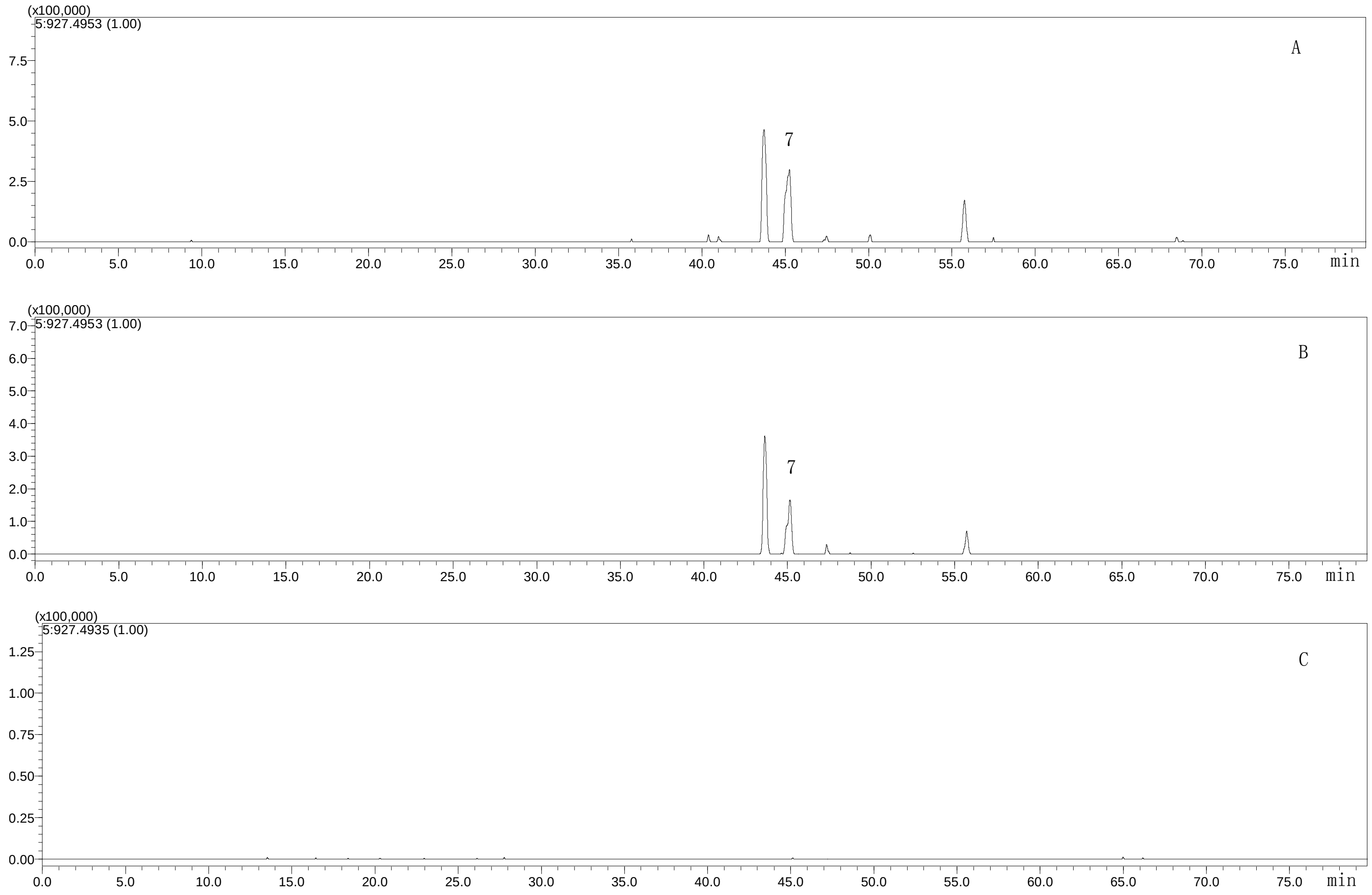

Figure S5. EIC chromatograms of compound **7** in negative mode at *m/z* 927.4953 from *I. pubescens* roots extract (A), ultrafiltrate (B) and blank sample (C).

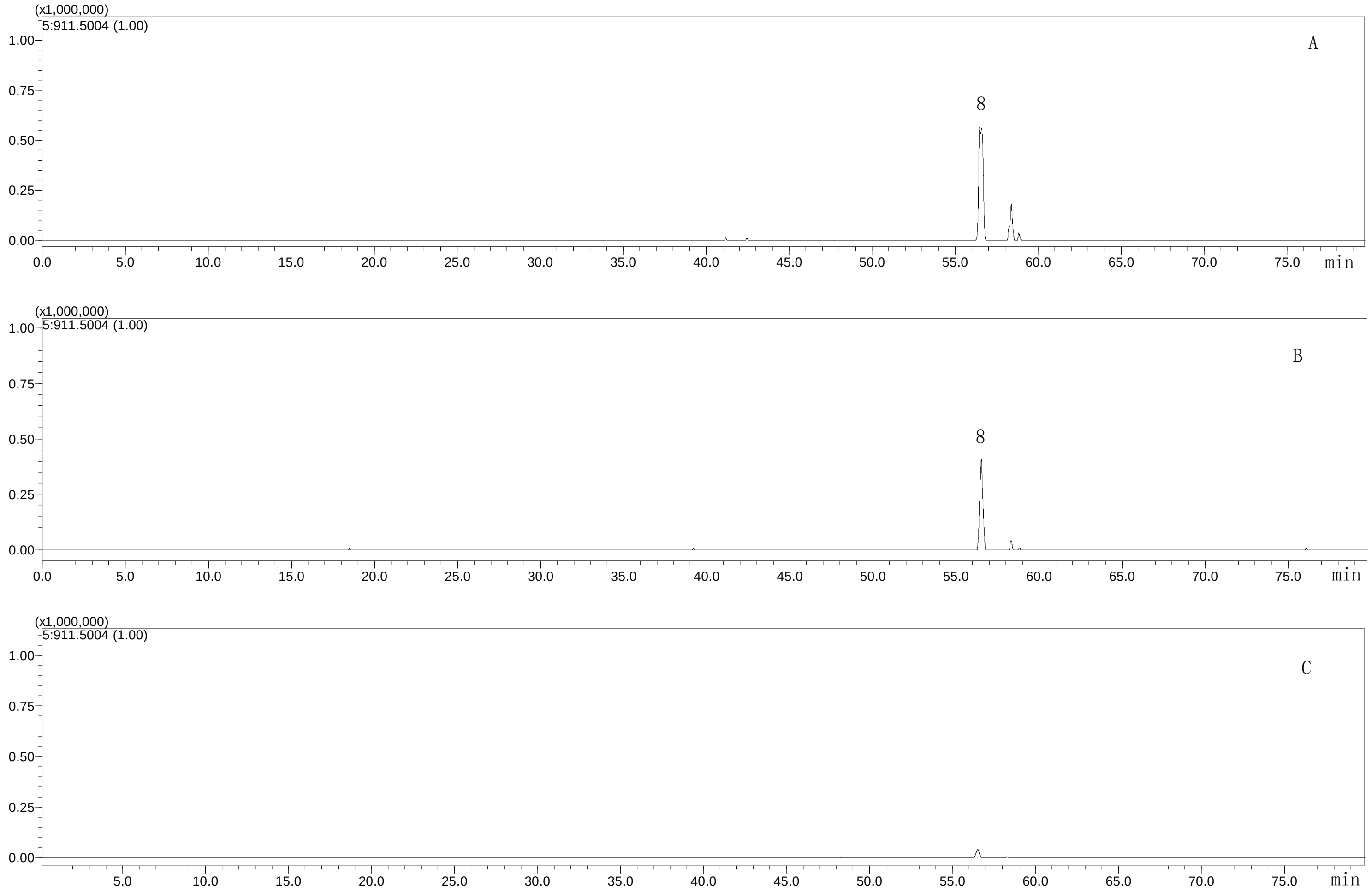

Figure S6. EIC chromatograms of compound **8** in negative mode at *m/z* 911.5004 from *I. pubescens* roots extract (A), ultrafiltrate (B) and blank sample (C).

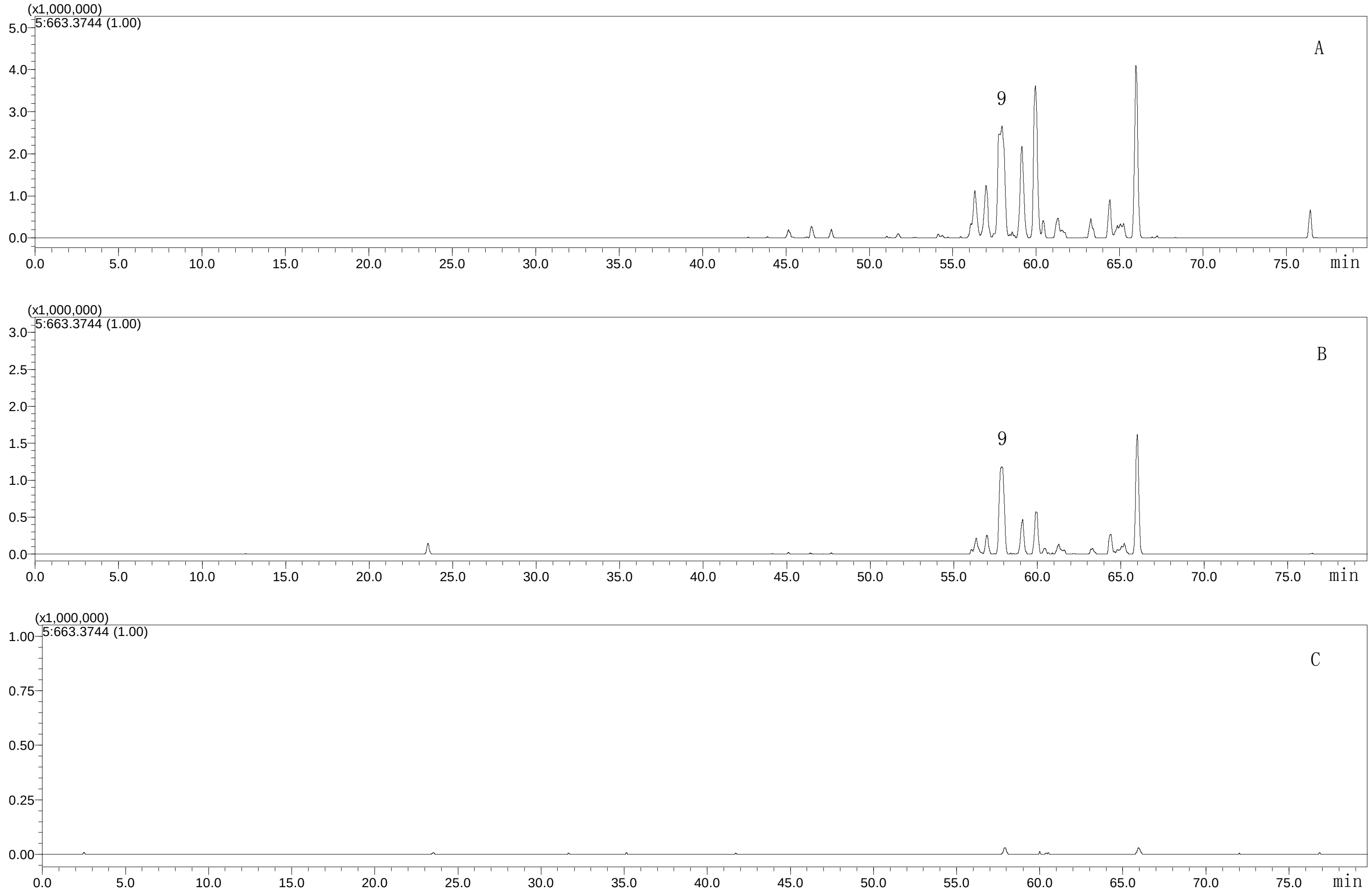

Figure S7. EIC chromatograms of compound **9** in negative mode at *m/z* 663.3744 from *I. pubescens* roots extract (A), ultrafiltrate (B) and blank sample (C).

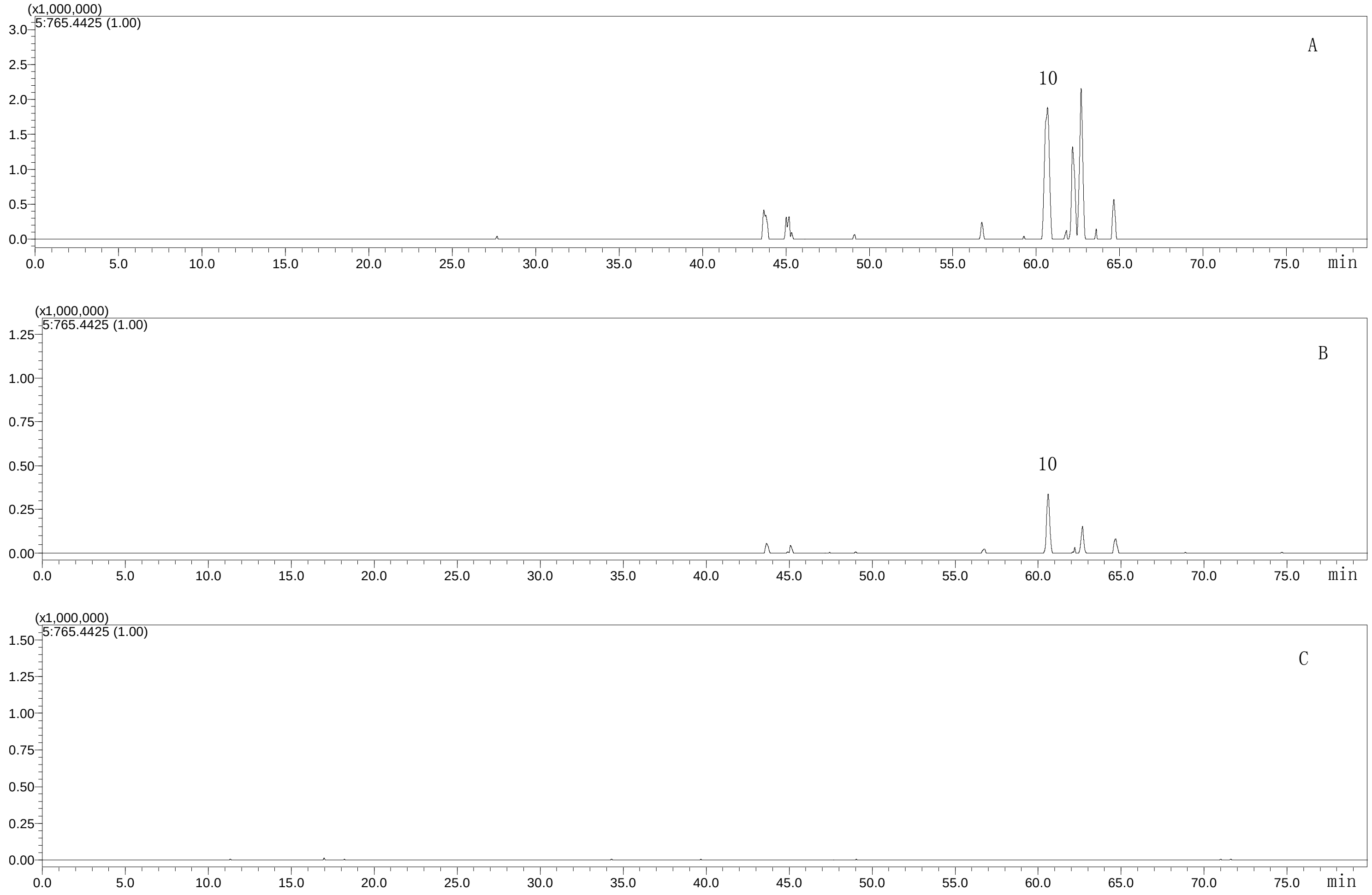

Figure S8. EIC chromatograms of compound **10** in negative mode at *m/z* 765.4425 from *I. pubescens* roots extract (A), ultrafiltrate (B) and blank sample (C).

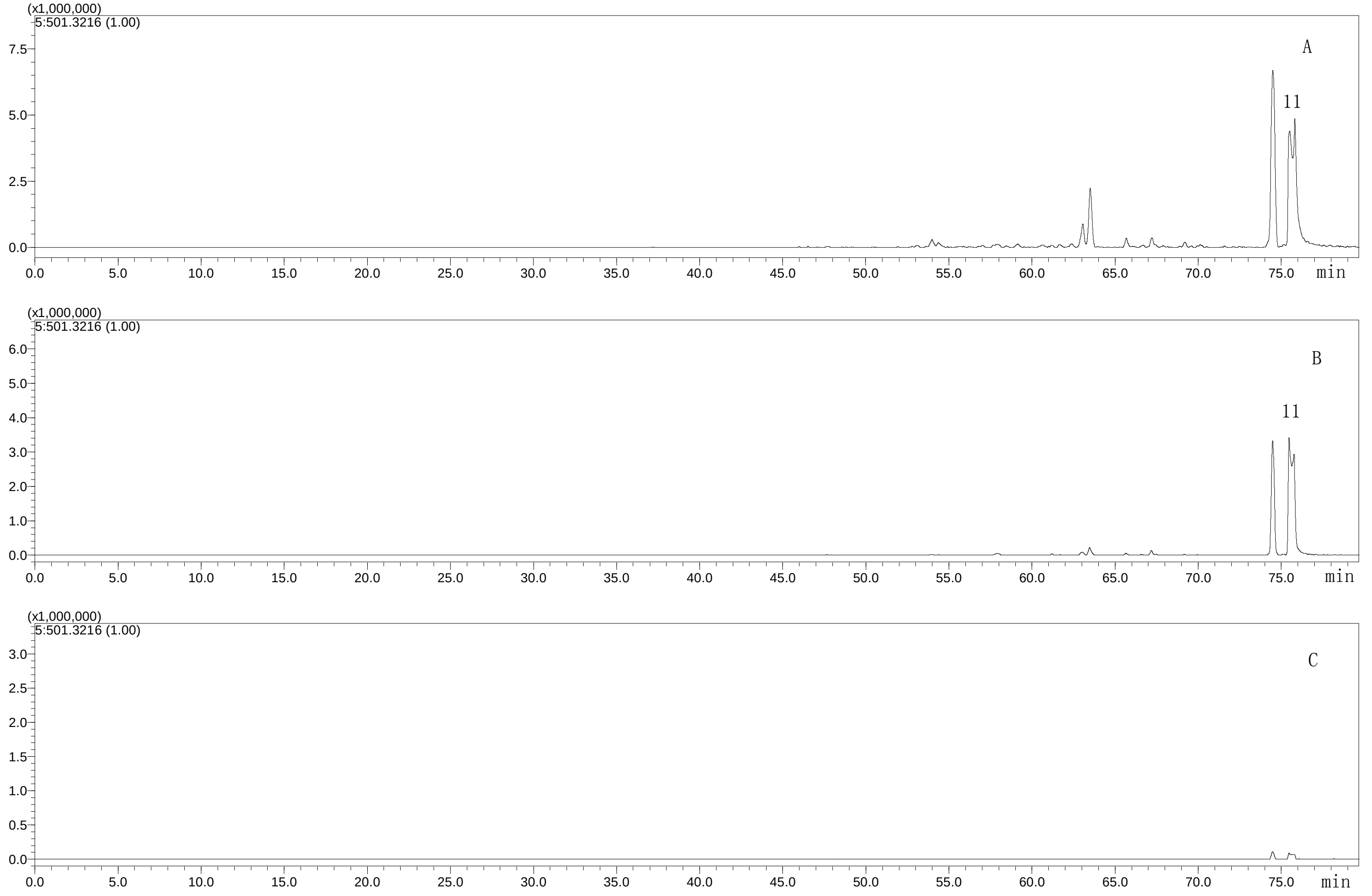

Figure S9. EIC chromatograms of compound **11** in negative mode at *m/z* 501.3216 from *I. pubescens* roots extract (A), ultrafiltrate (B) and blank sample (C).
